# Subcellular resolution three-dimensional light-field imaging with genetically encoded voltage indicators

**DOI:** 10.1117/1.NPh.7.3.035006

**Published:** 2020-08-28

**Authors:** Peter Quicke, Carmel L. Howe, Pingfan Song, Herman V. Jadan, Chenchen Song, Thomas Knöpfel, Mark Neil, Pier L. Dragotti, Simon R. Schultz, Amanda J. Foust

**Affiliations:** a Imperial College London, Department of Bioengineering, London, United Kingdom; b Imperial College London, Centre for Neurotechnology, London, United Kingdom; c Imperial College London, Department of Electrical and Electronic Engineering, London, United Kingdom; d Imperial College London, Department of Brain Sciences, London, United Kingdom; e Imperial College London, Department of Physics, London, United Kingdom

**Keywords:** light-field microscopy, genetically encoded voltage indicator, voltage imaging

## Abstract

**Significance:** Light-field microscopy (LFM) enables high signal-to-noise ratio (SNR) and light efficient volume imaging at fast frame rates. Voltage imaging with genetically encoded voltage indicators (GEVIs) stands to particularly benefit from LFM’s volumetric imaging capability due to high required sampling rates and limited probe brightness and functional sensitivity.

**Aim:** We demonstrate subcellular resolution GEVI light-field imaging in acute mouse brain slices resolving dendritic voltage signals in three spatial dimensions.

**Approach:** We imaged action potential-induced fluorescence transients in mouse brain slices sparsely expressing the GEVI VSFP-Butterfly 1.2 in wide-field microscopy (WFM) and LFM modes. We compared functional signal SNR and localization between different LFM reconstruction approaches and between LFM and WFM.

**Results:** LFM enabled three-dimensional (3-D) localization of action potential-induced fluorescence transients in neuronal somata and dendrites. Nonregularized deconvolution decreased SNR with increased iteration number compared to synthetic refocusing but increased axial and lateral signal localization. SNR was unaffected for LFM compared to WFM.

**Conclusions:** LFM enables 3-D localization of fluorescence transients, therefore eliminating the need for structures to lie in a single focal plane. These results demonstrate LFM’s potential for studying dendritic integration and action potential propagation in three spatial dimensions.

## 1 Introduction

Cellular resolution voltage imaging enables direct observation of neuronal computation. Indeed, membrane potential imaging experiments have spatiotemporally resolved both active and passive action, and synaptic potential generation throughout dendritic and axonal arbors.^1^^–^^14^ Resolution of these small voltage signals at high speeds requires high photon fluxes, making wide-field single-photon (1P) imaging by far the most common voltage imaging modality. Imaging neuronal processes with this technique require the imaged membranes to lie approximately flat in the microscope’s focal plane. As these experiments are typically performed in slices, the requirement for flat, healthy, and superficial cells represents a significant barrier to entry for experimenters. Even in the best-prepared slices, anatomy dictates that only a few cells will be oriented parallel to the surface, reducing experimental throughput, and only certain cell types feature morphology that can be well-sampled by a single plane. Iterative three-dimensional (3-D) imaging in wide field via physical refocusing is often untenable due to rapid sample bleaching. Multiple approaches to improve wide-field imaging’s 3-D performance have been developed. Anselmi et al.^15^ applied remote focusing to axially shift and tilt the wide-field focal plane as required by the sample, enabling calcium imaging along tilted dendrites. This adaptation, however, costs half of the fluorescence emission and is limited to a single tilted plane at a time. Point spread function (PSF) engineering via cubic phase masks^16^ or spherical aberration^17^ also enables parallelized volumetric sample imaging when combined with light-sheet excitation; however, to our knowledge, these approaches have not successfully been implemented to image membrane voltage.

Lack of optical sectioning with wide-field 1P imaging further complicates matters. Light from out-of-focus structures pollutes in-focus signals, confounding allocation of signals to axially separated processes. This issue is difficult to resolve with traditional optically sectioning confocal or two-photon microscopy approaches as they are point scanning. Sequential sampling of each pixel greatly reduces imaging bandwidth, and the fast frame rates required for voltage imaging necessitates short dwell times and, therefore, few collected photons. This restricts Poisson-noise limited signal-to-noise ratio (SNR) to low levels, making point scanning voltage imaging applicable to a limited number of experimental paradigms.^12^^,^^18^^–^^20^

Fluorescence excitation parallelization with multiple spots,^21^^–^^27^ spinning disks,^28^^,^^29^ blobs,^30^^,^^31^ lines,^32^^–^^34^ sheets,^35^^–^^42^ or specified patterns^43^^–^^47^ increases the photon budget, enabling functional volumetric imaging or single-plane imaging at increased speeds. A small number of these have been applied to imaging voltage in two dimensions,^26^^,^^33^^,^^44^^,^^47^ however, they are not able to image neuronal processes in 3D. Many of these techniques also trade-off reduced robustness to scattering compared to single-point scanning modalities for the increased excitation from parallelization.

Parallelized 3-D two-photon imaging with elongated Bessel^48^^,^^49^ or stereoscopic tilted^50^ beams excites narrow columns of fluorescence and relies on temporal and spatial sparsity of labeling and activity to demix time courses from different z planes. This increases the volume rate but each columnar pixel is still addressed sequentially, limiting bandwidth. These techniques have been used to image calcium fluorescence transients but not yet voltage.

**Fig. 1:**
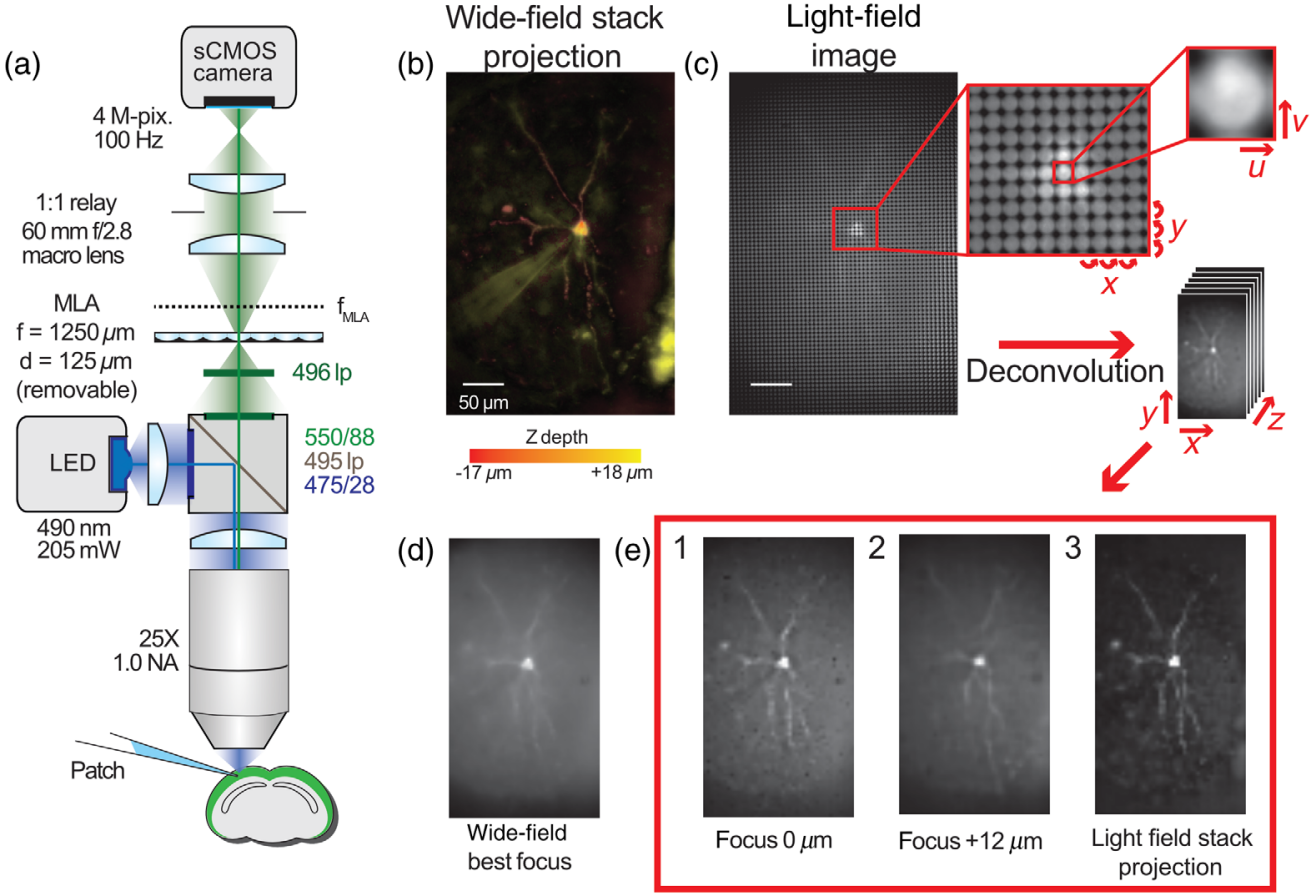
Light-field microscopy enables simultaneous focusing on axially separated dendrites. (a) LFM diagram. (b) A pseudocolor z-projection of a wide-field image stack through a GEVI labeled cell. Depth is color coded from red (superficial) to yellow (deep). Individual dendrites follow tortuous paths in all three dimensions, so they cannot be focused on simultaneously in WFM. (c) A light-field image of the same cell showing the structure of light-field images. Each spot in the light-field image is a spatial sampling (coordinates x, y) of the angular distribution of rays (coordinates u, v) at that point. This angular and spatial information can be used to reconstruct a volume from a single image. (d) A best focus wide-field image of the single cell showing partially in-focus dendritic structures. (e) Three different images recovered from the light-field image: (e1) and (e2) are the single axial planes deconvolved showing individual dendrites seen out-of-focus in the wide-field image. (e3) A z-projection through the recovered light-field volume image showing the in-focus sections of recovered dendrites. Figure adapted from Quicke^52^ CC BY-SA 4.0.

Light-field microscope (LFM)^51^ enables reconstruction of 3-D volumes from single two-dimensional (2-D) camera images, extending wide-field imaging while maintaining its unparalleled fluorescence excitation and collection efficiency. This is achieved by inserting a microlens array (MLA) at the native image plane of the microscope and placing the image sensor at its back focal plane [Fig. 1(a)]. This disperses the angular components of the collected image [Fig. 1(b)], which can be used to infer objects’ axial positions. Each LFM image consists of circular subimages [Fig. 1(c)], with each subimage resembling a pixel in an undersampled image of the scene. Within each circular subimage, each pixel location encodes a different angular sampling through the object intersecting with the subimage’s location, a columnar tomographic projection through the sample.^53^ Light-field images are typically parameterized by the four-dimensional (4-D) function L(u,v,x,y), where each lenslet subimage is L(u,v,·,·) and the same specific pixel under each subimage is L(·,·,x,y). The “native LFM resolution” with which the object is laterally sampled is given by the microlens pitch divided by the objective magnification, much worse than the corresponding wide-field resolution. In exchange, the microlenses provide angular information that can be used to render views of the object from different perspectives, focus on different planes, and reconstruct 3-D volumes all from a single 2-D frame. This technique converts a key disadvantage of wide-field 1P fluorescence excitation, lack of optical sectioning, into an advantage, as out-of-focus light renders 3-D information about the sample.

Two prominent algorithms for reconstructing source volumes from LFM images have been described, namely synthetic refocusing^51^ and 3-D deconvolution.^53^ Synthetic refocusing relies on a ray optics model of LFM image formation to reconstruct images at the native LFM resolution equivalent to those of a wide-field microscope (WFM) focused at any axial plane in the sample. Focal stacks can be generated similar to standard microscope z-stacks by combining images reconstructed at multiple axial depths. Each pixel in the refocused image is a weighted sum of light-field image pixels, meaning refocusing is fast. The reconstructed images, however, suffer from the same blur due to lack of optical sectioning as a standard WFM.

An alternative approach is based on reconstruction of the source volume using a forward model of light-field image formation (the LFM PSF) based on wave optics.^53^ Iterative deconvolution approaches, such as Richardson–Lucy (RL)^54^^,^^55^ or the image space reconstruction algorithm (ISRA),^56^ find the maximum likelihood source volume given the measured image and LFM PSF in the presence of Poissonian (RL) or Gaussian (ISRA) noise. This approach can reconstruct source volumes at a lateral resolution greater than the native LFM resolution (the MLA pitch in the sample) by leveraging the fine sampling of the LFM tomographic projections.^53^ This increased resolution reconstruction fails where the tomographic sampling is degenerate, most notably around the native focal plane of the microscope. The source volume is also reconstructed with less axial blur than in the refocused case, increasing axial signal discriminability.

Newer light-field designs have significantly improved LFM performance and circumvented the degenerate sampling at the native focal plane. High-resolution LFM displaces the degenerate sampling away from the native focal plane, enabling higher resolution object reconstruction^57^. Cohen et al.^58^ removed the sampling degeneracy and improved the reconstruction via placing a phase mask in the objective back focal plane. Particularly important, Fourier LFM^59^^,^^60^ modifies the LFM design to place the MLA in the Fourier plane of the microscope. This design eases reconstruction computational cost due to its laterally invariant PSF and also does not suffer from reconstruction issues around the native focal plane. However, the PSF periodicity does result in reconstruction artifacts necessitating a reduction in the field of view (FOV) to remove. Recently, Liu et al.^61^ further improved on LFM with a particularly promising design, removing reconstruction artifacts using a random MLA, and increasing the reconstructable depth of field using microlenses of different focal lengths.

Electrical length constants in neurons are on the scale of tens to hundreds of microns, making increased lateral pixel size less disadvantageous for voltage imaging. Over-resolving electrical fluctuations by imaging at or below the diffraction limit are typically unnecessary and can even hurt SNR by increasing the relative impact of non-Poisson noise, such as read noise. Spatial resolution is therefore often sacrificed in voltage imaging experiments to increase speed or SNR. Many such experiments use low read noise, high sensitivity CCD sensors featuring low pixel counts, with pixels often measuring several microns across in the sample plane. Even with higher pixel count detectors, the relatively low sensitivity of many voltage probes means multiple pixels are often binned to increase SNR to acceptable levels. Therefore, LFM’s decreased native lateral sampling rate suits voltage imaging well, and deconvolution of LFM voltage imaging time series can be implemented without oversampling to reduce computational cost.

LFM has successfully imaged calcium over large volumes in *C. elegans* and zebrafish,^62^^,^^63^ and in both head-fixed and behaving mice.^64^^–^^66^ Voltage dynamics have also been imaged successfully without single-cell resolution in *Drosophila*^67^ and larval zebrafish^60^ as part of whole-brain imaging setups alongside calcium imaging. LFM has not, to our knowledge, been applied to studying subcellular or single-cell resolution voltage dynamics in any sample, despite its apparent suitability. In this study, we apply LFM to subcellular genetically encoded voltage indicator (GEVI) imaging in acute mouse brain slices. We combine this technique with a recently reported transgenic strategy driving sparse expression in a random subset of layer 2/3 cortical pyramidal neurons, which enables the resolution of single-cell level voltage signals in neuronal somata and dendrites.^68^^,^^69^

We demonstrate that LFM can simultaneously image axially separated dendrites, enabling single-shot capture and localization of GEVI fluorescence transients in the 3-D dendritic arbor. We compare and evaluate deconvolution and synthetic refocusing for different GEVI imaging applications, while using a coarse deconvolution approach with no lateral oversampling to reduce computational cost. We also apply a recently developed LFM PSF calculation^70^ for high-NA objectives. We show that LFM enables 3-D localization of dendritic and somatic GEVI fluorescence transients and compare the extent to which refocused and deconvolved light fields enable lateral and axial transient localization. Finally, we compare temporal signal SNR between LFM and WFM.

## 2 Methods

This section reproduces methods described by Quicke.^52^ We designed our LFM following the principles set out by Levoy et al.^51^ We adapted a wide-field imaging system by placing an MLA at the microscope image plane and used a 1:1 relay lens (Nikon 60-mm f/2.8 D AF Micro Nikkor Lens) system to image the MLA back focal plane onto our camera chip [ORCA Flash 4 V2, 2048×2048  pixels, 6.5-μm pixel size, Hamamatsu, see Fig. 1(a)]. The lateral resolution is given by the MLA pitch divided by the magnification of the objective. Using our 25× objective (1.0 NA, XLPLN25XSVMP, Olympus), we chose our system to have 5-μm lateral pixels, dictating a microlens pitch of 125  μm.

The axial resolution is defined by the number of resolvable diffraction-limited spots behind each microlens.^51^ Assuming a central emission wavelength of 550 nm for mCitrine, the förster resonance energy transfer (FRET) donor in VSFP-Butterfly 1.2,^71^ the spot size in the camera plane is 6.46  μm using the Sparrow criterion. With a 125-μm pitch MLA, we can resolve $N_{u}=19$ distinct spots under each microlens. The axial resolution when synthetically refocusing our LFM can, therefore, be calculated as 7.81  μm.^51^

To efficiently use the camera sensor, the exit pupil of the objective should map through the MLA to produce circles on the light-field plane that are just touching, requiring that the objective image-side f-number (f/12.5) equal the MLA f-number. We chose an f/10 MLA (MLA-S125-f10, RPC Photonics), an off-the-shelf part that came close to matching while being a larger aperture.

### 2.1 Imaging

This study was carried out in accordance with the recommendations of the UK Animals (Scientific Procedures) Act 1986 under Home Office Project and Personal Licenses (project licenses 70/7818 and 70/9095). Slices were made from four mice aged 31, 32, 32, and 175 days transgenically modified to sparsely express VSFP-Butterfly 1.2^71^ using the method previously described by Quicke et al.^68^ and Song et al.^69^ These transgenic mice express the GEVI in cortical layer 2/3 pyramidal neurons under the intersectional control of TetO and destabilized Cre-recombinase.^72^^–^^74^ The destabilized Cre-recombinase was stochastically restabilized to induce sparse expression of the voltage indicator via two IP injections of a total of $2\times 10^{-4}\;\mathrm{mg}\;{\mathrm{kg}}^{-1}$ Trimethoprim (TMP, Sigma) over two consecutive days as described by Song et al.^69^

Slices were prepared at least 2 weeks post-TMP injection using a method adapted from Ting et al.^75^ (the “protective recovery” method^76^). Slices of 400  μm were cut with a Camden Microtome 7000 in ice cold 95% $O_{2}$/5% ${\mathrm{CO}}_{2}$ oxygenated artificial cerebrospinal fluid (ACSF) containing: (in mM) 125 NaCl, 25 ${\mathrm{NaHCO}}_{3}$, 20 glucose 2.5 KCl, 1.25 ${\mathrm{NaH}}_{2}{\mathrm{PO}}_{4}$, 2 ${\mathrm{MgCl}}_{2}$, and 2 ${\mathrm{CaCl}}_{2}$. The slices were then immediately transferred into NMDG-ACSF^75^ containing: (in mM) 110 N-methyl-D-glucamine, 2.5 KCl, 1.2 ${\mathrm{NaH}}_{2}{\mathrm{PO}}_{4}$, 25 ${\mathrm{NaHCO}}_{3}$, 25 glucose, 10 ${\mathrm{MgCl}}_{2}$, 0.5 ${\mathrm{CaCl}}_{2}$, adjusted to 300 to 310  mOsm/Kg, pH 7.3 to 7.4 with HCl and oxygenated with 95% $O_{2}/5\%$ ${\mathrm{CO}}_{2}$ at 36°C for 12 min before being transferred back into the original sodium-containing ACSF for at least an hour before patching and imaging.

Fluorescent cells were patched under oblique infrared illumination (780 nm) with pipettes of resistances between 3 and 10 Mohms when filled with intracellular solution containing: (in mM) 130 K-gluconate, 7 KCl, 4 ATP-Mg, 0.3 GTP-Na, 10 phosphocreatine-Na, and 10 HEPES is 4-(2-hydroxyethyl)-1-piperazineethanesulfonic acid. We digitized current clamp signals (Power 1401 digitizer; Cambridge Electronic Design) from a Multiclamp 700B amplifier (Axon Instruments). At room temperature, we imaged at 100  frames/second for 2.5 s while injecting current pulses lasting 50 and 100 ms. A 100-Hz imaging is sufficient for detecting action potentials with this voltage indicator due to the indicator’s slow kinetics. This GEVI, unlike recently developed indicators or organic voltage dyes, reports a low-pass filtered version of the membrane voltage signal. Second, as discussed by Quicke et al.,^68^ lower frame rates can be useful for low-SNR spike detection, as the signal is further low-pass filtered by the camera integration period, and the longer frame period allows collection of more photons, increasing shot-noise limited SNR. Higher frame rates would better resolve action potential timing and kinetics. Each pulse elicited depolarization to threshold evoking a single action potential or burst of two to three action potentials. We powered a 490-nm LED (M490L4, Thorlabs) with a constant current source (Keithley Sourcemeter 1401) to illuminate the sample at 3 to $11\;\mathrm{mW}/{\mathrm{mm}}^{2}$. Sets of light-field time series and wide-field time series acquisitions were interleaved by removing and replacing the MLA by hand. We averaged between 4 and 16 sweeps per imaging condition. The LED was collimated with an f=16-mm aspheric lens (ACL25416U0-A, Thorlabs) and filtered with a 475/28  nm excitation filter (FITC-EX01-CLIN-25, Semrock). Fluorescence was collected using a 495-nm long-pass dichroic (FF495-Di03, Semrock) along with a 550/88-nm collection filter (FF01-550/88, Semrock) and 496 long-pass filters (Semrock FF01-496/LP) to attenuate any excitation light transmitted by the dichroic. Imaging data were acquired with Micromanager.^77^ Imaged cells’ somata lay between 11 and 40  μm below the slice surface, with a median depth of 29  μm. These cells all lie within the photon mean free path of the slice surface,^78^ and our reconstruction methods do not take tissue scattering into account. Data were analyzed with custom Python scripts using SciPy packages.^79^

### 2.2 Light-Field Reconstruction

We reconstructed source volumes using two techniques to compare their performance for single-cell voltage data. We calculated (x,y,z,t) volume time series using synthetic refocusing,^51^ and ISRA^56^^,^^62^ using a PSF calculated using the method described in the section as follows. RL deconvolution^53^^–^^55^ was also tested on the data, however, little discernible difference in the results was observed.

#### 2.2.1 Light-field PSF calculation

We calculated LFM PSFs differently to previously described,^53^ using the method described by Quicke^52^ and Quicke et al.^70^ Briefly, to calculate the field at the MLA, we considered how a high-NA objective lens collects the field from an oscillating electric dipole at position r near the microscope focus, |r|≪f, at the origin, calculating the Fourier transform of the field in the objective back focal plane. We assumed that we could model the behavior of a point source consisting of randomly oriented fluorescent molecules as the incoherent sum of dipoles along three orthogonal directions. We then used the same method as described by Broxton et al.^53^ to model transmission through the MLA and to the camera.

We calculated the PSF for GEVI imaging deconvolution for 550-nm emission. We did not oversample the deconvolution as resolving voltage signals generally requires averaging pixels to approximately the native LFM resolution. We, therefore, generated a single light-field kernel for each depth by averaging over kernels sampled for point sources at different lateral positions under the microlens, weighting each point in the average by a 2-D Hamming window function of a width equal to our microlens’ pitch. We averaged over kernels sampled at five times finer than the native microlens resolution. The ISRA was used to deconvolve the data.

#### 2.2.2 Volume reconstructions

Having obtained our downsampled PSF, we deconvolved our volume using a similar procedure to previous studies. A key difference is that only a single 2-D convolution was required for each depth in the reconstructed volume for the forward and backward projections, respectively, as we did not increase the lateral sampling rate. We applied the deconvolution scheme independently to each frame of the image time sequences, using a cluster to parallelize the data processing. Deconvolution of a single frame took around 30 to 40 min for a 21 iteration deconvolution of 21 z planes on a single CPU. We employed a large cluster to process the individual frames simultaneously, enabling 5000 frames to be processed overnight. We did not use a parallel algorithm within each deconvolution to leverage, e.g., GPU processing, as the computing resources available to us were better suited to data parallelism. As with previous studies, this would greatly increase the rate of individual frames, although it would also likely reduce the number of simultaneous frames that could be deconvolved for typical cluster setups.

Synthetic refocusing, based on a ray optics model of light-field image formation, is a simpler approach to volume reconstruction that is also much less computationally intensive. Images focused at different z-depths can be constructed by combining individual perspective views using the formula derived by Ng et al.^80^ Linear interpolation in this summation results in each pixel being the weighted sum of pixels of the original light-field image. This reconstruction is much faster than the iterative deconvolution methods and also does not suffer from noise amplification.^81^

### 2.3 Volume Time Series Analysis

#### 2.3.1 Effect of reconstruction on SNR

To compare the effect of different reconstruction techniques on voltage signal SNR, we reconstructed single planes from volumes at the LFM focus. We compared synthetically refocused time series with time series deconvolved using ISRA for different iteration numbers. Regions of interest (ROIs) were manually chosen over the soma and its surround and were identical for the synthetically refocused and deconvolved volumes.

**Fig. 2:**
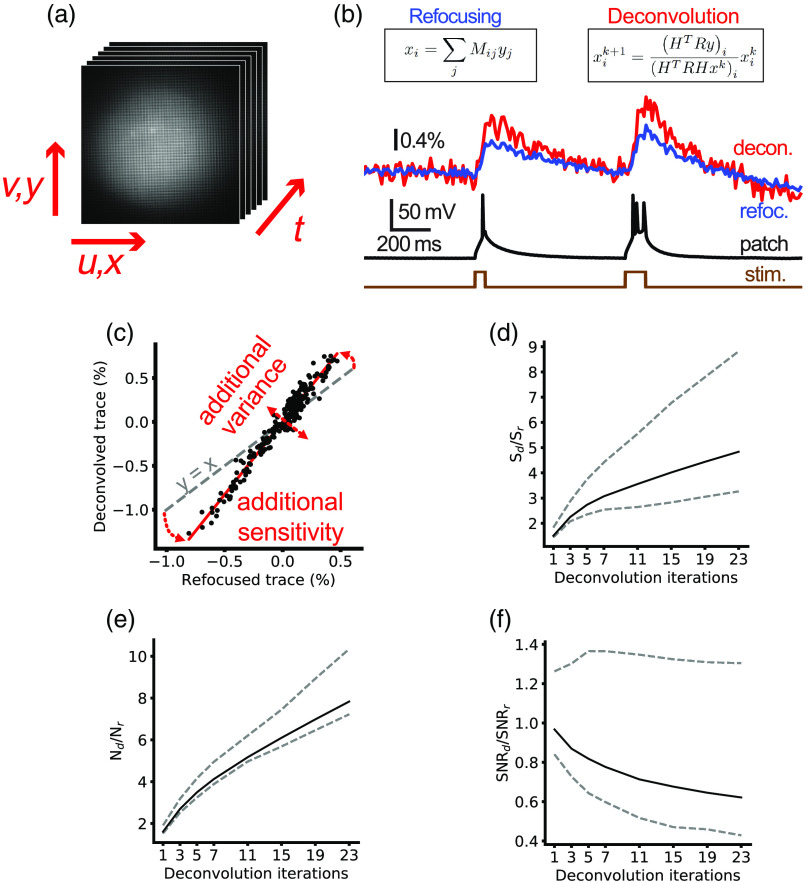
Comparison of different reconstruction methods on SNR. (a) Light-field time series were collected of functional voltage signals from sparsely expressed GEVIs. (b) Time series were extracted from in-focus image sequences of the soma via refocusing (left) and ISRA deconvolution (right) and the signal and noise were compared. (c) Deconvolved and refocused signals are strongly linearly correlated, as can be seen from plotting the individual trace time points. The additional noise variance due to deconvolution can be identified as the residual from the linear fit. The increased signal level can be seen as the increased fit gradient over unit slope (gray dashed line). Both the (d) noise and (e) signal increase monotonically with increasing deconvolution iteration, leading to an overall reduction in (f) SNR with iteration number. At low iteration number, deconvolution and refocusing are very similar. At large iteration number, the SNR is decreased relative to refocused; however, increased axial sectioning may still motivate the use of deconvolution methods. Solid lines are median of n=15 cells and dashed lines indicate 25th and 75th percentile values. Traces in (b) were generated from an average of eight sweeps.

**Fig. 3:**
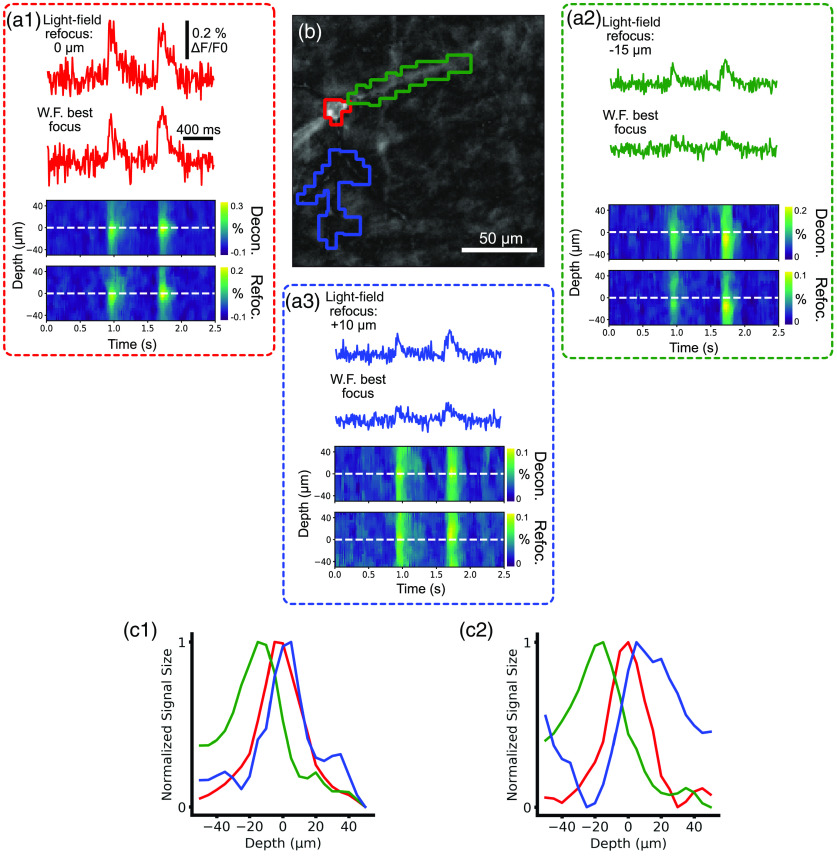
Deconvolution LFM resolves 3-D localized voltage signals. (a1)–(a3) Time courses and depth-time plots showing signals from different cellular compartments [shown in (b)] localized at different depths. (a1) The somatic signal is maximal in the wide-field and native light-field focal planes, while (a2) the apical dendrite descends into the slice with its ROI localized 15  μm deeper. The signal from (a3) a basal dendrite is superficial to the soma, and its best focal depth is difficult to localize due to the broad axial extent of the refocused signal. The basal and apical dendritic fluorescence transients in the wide-field time courses have smaller signals than the light-field signals as they are out-of-plane when focused on the soma. (c) The normalized signal size for each ROI across different (c1) deconvolved and (c2) refocused depths. Deconvolution increases the axial localization of signals. The data are an average of eight sweeps.

As we were collecting fluorescence from the VSFP-Butterfly 1.2 FRET donor, fluorescence decreased upon membrane depolarization.^71^ Therefore, the traces shown Figs. 2 and 3 are inverted. To measure SNR, we calculated the signal as the fifth percentile value during a stimulus and relaxation period of 200 ms with the median value of the 100 ms before the stimulation period subtracted. The noise level was calculated as the standard deviation of a 350-ms period during no intracellular current stimulus.

#### 2.3.2 Depth-time plots

To determine the center of mass of the signal for different cellular ROIs, we extracted time courses from each refocused or deconvolved depth, then filtered the resulting depth-time 2-D arrays with a median filter of 11 samples in the time axis (110 ms) and three samples in the z axis (15  μm).

#### 2.3.3 Comparison of light-field and wide-field SNR

We compared the SNR between trials of the same cell for image sequences taken with wide-field and light-field imaging systems. We compared the SNR between refocused and wide-field images for the same number of repeats using ROIs calculated to be the same for both imaging modalities. For 8/12 cells, an extra aperture was introduced into our LFM to compensate for chromatic aberration, reducing the light throughput of the microscope by between 1/2 and 3/4 during light-field imaging compared to the equivalent wide-field trials. To account for this, the SNR for these trials was adjusted by a factor equal to the square root of the ratio of the mean brightness of the first imaging trials from the light-field and wide-field trials. The microscope was realigned to account for chromatic aberration before the final 4/12 cells, which means that the design light throughput of the microscope was the same between light-field and wide-field trials. For these trials, the raw SNR was included in the analysis.

#### 2.3.4 Signal spread analysis

We compared the lateral and axial signal spread using a method similar to our previous work.^68^ We quantified the neuronal voltage signal strength in each pixel to create 2-D or 3-D “activation maps” by calculating the temporal correlation coefficient of each pixel’s time course with a seed time course from the somatic ROI.

We compared the spatial autocorrelations of these activation maps to quantify the average signal crosstalk between cellular voltage signals.^68^ In our previous work, we described how the autocorrelation can be used to quantify the average signal power a cell contributes to a specific pixel’s time course and quantified this effect for this preparation with WFM in two dimensions.^68^ In this work, we calculated the 3-D autocorrelations of the light-field volumes and 2-D autocorrelations of the wide-field volumes using Fast Fourier transform (FFT)-based convolution, setting the central 10×10  pixels of the autocorrelations to the mean of their perimeter to remove a central noise peak.

## 3 Results

### 3.1 Light-Field Microscopy Enables Simultaneous Imaging of Axially Separated Dendrites.

We demonstrated LFM’s ability to resolve axially separated structures by imaging a cell with a complex 3-D dendritic arbor using both WFM [Fig. 1(b)] and LFM [Fig. 1(c)]. No single plane wide-field image was able to simultaneously bring all the dendrites into a good focus [Fig. 1(d)]; however; in different planes from a volume reconstructed by deconvolution different dendritic structures could be clearly distinguished [Figs. 1(e1) and 1(e2)]. The same cellular features can clearly be seen in a standard deviation projection through the reconstructed LFM stack [Fig. 1(e3)] and a wide-field z stack through the same cell [Fig. 1(b)], both projections through stacks at 1-μm axial increments].

### 3.2 Comparison of the Effect of Different Reconstruction Methods on Signal-to-Noise Ratio

Low-sensitivity GEVIs mean SNR is of utmost importance in voltage imaging analysis strategies, and so we first compared the performance of the deconvolution and refocusing reconstruction approaches on this metric. We reconstructed the volume time series for 15 cells from light field (LF) time series [Fig. 2(a)] and extracted optical time courses from ROIs over the individual cell’s soma at the native focal plane and compared the SNR between deconvolved and refocused volumes [Fig. 2(b)]. Commonly used LFM iterative reconstruction schemes are prone to noise amplification^82^ which increases with iteration number. It is therefore crucial to understand when to stop the iteration scheme. Early stopping provides a regularizing effect on the deconvolution scheme, reducing noise contamination in the final reconstruction at the cost of model fidelity.^83^ We used the refocused images as a baseline comparison for the iteration analysis due to the ease of their reconstruction. We found that for all iteration numbers, the noise and signal level were increased by deconvolution which increased sensitivity and variance [Figs. 2(c)–2(e)]. The signal significantly increased from 0.3% (0.2%, 0.4%) [all results presented as median interquartile range (IQR)] in the refocused time series to 1.4% (0.9%, 1.7%) for the 21 iteration deconvolved traces (Wilcoxon signed rank, n=15, z=0.0, and p=0.0003). The noise significantly increased from 0.05% (0.04%, 0.08%) in the refocused time series to 0.4% (0.3%, 0.5%) for the 21 iteration deconvolved traces (Wilcoxon signed rank, n=15, z=0.0, and p=0.0002). This resulted in the SNR reducing from approximately the same as the refocused [1.0 (0.8, 1.3)] case for a single deconvolution iteration to around half that of the refocused case [0.6, (0.4, 1.3)]. We also processed the light-field time series using RL deconvolution and found no substantial differences compared to ISRA.

### 3.3 Light-Field Microscopy Resolves 3-D Localized and Axially Separated Voltage Signals

We then explored a key advantage of subcellular resolution light-field voltage imaging: 3-D imaging of neuronal processes. Achieving this requires signals from different planes to be discriminable in volume reconstructions. Axial discriminability depends on intrinsic factors, such as axial resolution, and also extrinsic factors, such as cellular morphology and signal spread due to tissue scattering. To demonstrate the resolution of subcellular voltage transients in 3D, we reconstructed 4-D (x,y,z,t) volumes from light-field image time series and compared the temporal signals from ROIs over different dendritic and somatic structures in multiple axial planes.

Figure 3 demonstrates LF imaging’s ability to axially localize functional voltage signals from neuronal processes and thereby image functional activity in 3D with SNR unachievable by any equivalent wide-field system. Figures 3(a1)–3(a3) show single plane and multiplane time courses from three different ROIs over cellular compartments from a neuron distributed over multiple axial planes. A somatic ROI [Fig. 3(a1)] in the native focal plane of both wide-field and light-field images contains action potential evoked fluorescence transients approximately equal in signal size for both light field and wide field (top). The depth-time plots show the functional signal localized to the native focal plane in the LF functional stacks (bottom). In contrast, an ROI over the apical dendrite [Fig. 3(a2)] has the largest signal when the LF image is refocused 15  μm deeper into the slice, and the signal in the equivalent wide-field ROI is much smaller. The depth-time plots for this ROI from both deconvolved and refocused stacks also clearly show the center of mass of the signal located deeper than the native focal plane [Figs. 3(c)1 and 3(c)2, bottom]. Signals from a basal dendrite [Fig. 3(a3)] are similarly larger in the LF image refocused 10  μm shallower than the native focal plane. The corresponding depth-time plots show a slight shift in the signal center of mass to a shallower depth, especially in the refocused case.

Plots of signal size as a function of depth for the refocused and deconvolved cases [Figs. 3(c1) and 3(c2)] show the axial localization as distinctly different planes for each ROI and also demonstrate a key advantage of deconvolved over refocused reconstructions: increased accuracy in axial localization of functional signals.

### 3.4 Deconvolution Increases Axial Localization of Functional Voltage Signals

**Fig. 4:**
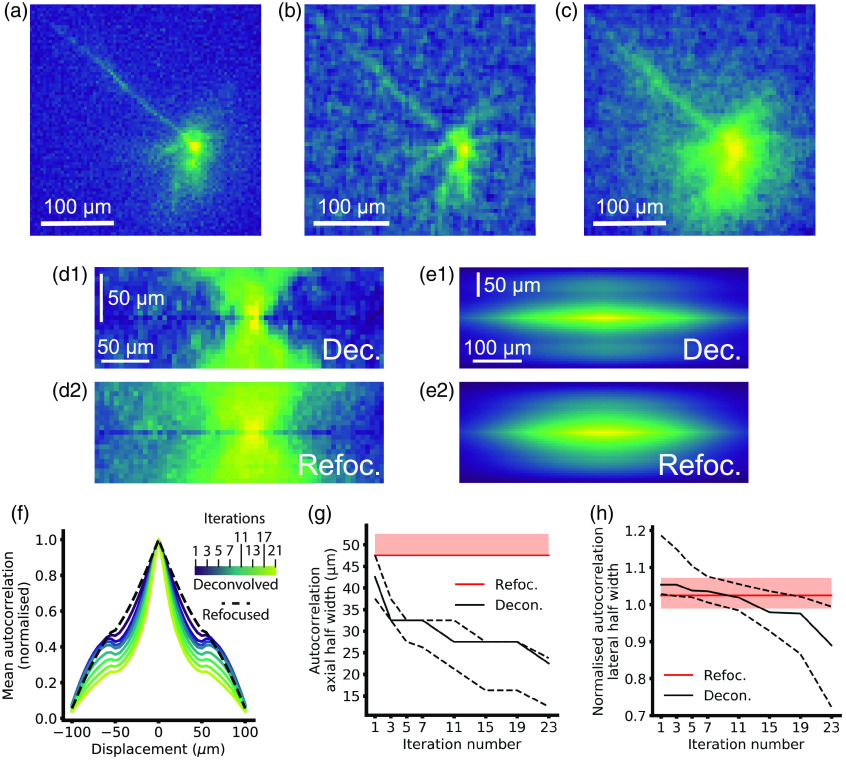
Mapping dendritic signals. (a) Wide-field “activation” image. Yellow pixels contain large voltage signals, while blue pixels contain low or no voltage signals. (b) Deconvolved activation image, sum projection from −35 to +35  μm, five deconvolution iterations. (c) Refocused activation image, sum projection from −35 to +35  μm. (d) xz maximum intensity projections through (d1) deconvolved, and (d2) refocused activation images showing the different axial sectioning. (e) Mean xz projections through the autocorrelations. (f) Normalized maximum autocorrelation for different depths from refocused and deconvolved LFM activation volumes. The secondary peaks arise from the elongated axial PSF, and these can be seen decreasing as the iteration number increases. (g) Median autocorrelation axial half widths for n=12 cells with iteration number. Dashed lines represent quartile values. Red line is the refocused median width and shaded area of the refocused IQR. (h) Median autocorrelation lateral widths normalized to wide-field lateral widths for refocused images (red and shaded area IQR) and different deconvolution iterations (black lines and dashed lines IQR).

The transients from refocused volumes exhibit a larger axial PSF width compared to the deconvolved traces [Fig. 3(c)]. Hence, these signals are smeared out, reducing distinguishability of signal contributions from different planes. To quantify this effect, we generated volumes showing the distribution of functional signal. We generated a time course from an in-focus somatic ROI and calculated the temporal correlation coefficient of every pixel in the volume for refocused and deconvolved volume time series. Pixels with high correlation coefficients are interpreted as having a large response to the intracellular current injection, and so a volume map of these reveals morphology of structures through which the functional signal propagates. These activation maps of voltage signal (Fig. 4) enabled us to quantify the spatial signal distribution from the same cells with different imaging techniques. Activation maps from wide-field imaging trials show blurring around the soma from out-of-focus basal dendrites [Fig. 4(a)]. Comparatively, z projections from a 70-μm region around the soma generated from the deconvolved activation volume [Fig. 4(b)] reveals the structures that cause this blur. A projection through 70  μm around the focus from the refocused case shows significantly more blurring due to the poor axial sectioning of this technique [Fig. 4(c)]. We used the 3-D autocorrelation of these activation maps to quantify the spread of the signal in 3D (see Sec. 2.3.4). Measuring the autocorrelation of the activation maps provides a convenient method for comparing the average voltage signal lateral and axial width between reconstruction approaches, as complex structures distributed in 3D are automatically “aligned.” The autocorrelation of the cellular signal can also be used to quantify the contribution of signal crosstalk from an adjacent cell to nearby pixels.^68^ To this end, we first quantified how the peak autocorrelation from each cell, and therefore functional signal contribution, decayed axially. Axial smearing can be seen in reconstructions from both deconvolution and refocusing [Fig. 4(d)], although the effect is much more severe in refocused traces. The smearing appears in the axial autocorrelation as both broadening of the central peak and increased side lobes [Fig. 4(e)]. The central peak width and side lobes decrease with increased deconvolution iteration number [Figs. 4(f) and 4(g)], thus increasing the axial sectioning.

The autocorrelation widths decreased significantly from 1 to 21 iterations [median dropped from 42.5 (37.5, 47.5) to 22.5 (12.5,23.75)  μm, z=0, and p=0.002], and for both cases, the axial spread was significantly lower than refocused [median of 47.5 (47.5,52.5)  μm, p=0.001, and p=0.002 for 1 and 21 iterations, respectively]. Significance tests were performed with a Friedman $\chi^{2}$ with post hoc Bonferroni-corrected Wilcoxon signed-rank tests (significant at p<0.017). Here, n=12 cells from 12 slices from four mice. Friedman $\chi^{2}=24$ and $p=6\times 10^{-6}$.

**Fig. 5:**
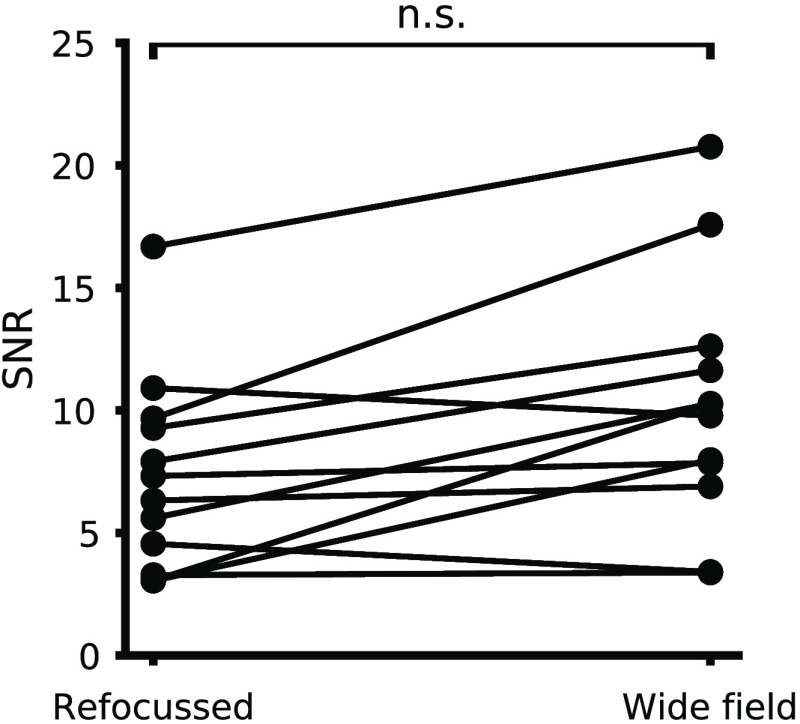
Comparison of light-field SNR and wide-field SNR. Points correspond to mean SNR between paired light-field and wide-field trials. LFM SNR does not differ significantly from wide field. For 8/12 trials, we included a correction factor due to a misalignment in the LFM as discussed in Sec. 2.3.3.

Finally, we compared how deconvolution and refocusing affected lateral signal localization compared to the equivalent wide-field time series [Fig. 4(h)]. We measured the width of radially averaged autocorrelations normalized to matched wide-field trials for the refocused and deconvolved cases. We found that the lateral signal spread significantly decreased from 1 to 21 iterations [median dropped from 1.05 (1.03, 1.18) times larger than wide-field trials to 0.89 (0.73, 0.99) times larger, Z=0, p=0.002], from significantly larger than the wide field and refocused at 1 iteration (z=1, p=0.003 and z=0, p=0.002, respectively), to significantly smaller than the refocused at 21 iterations (z=0.0, p=0.003). The refocused widths did not differ significantly from the matched wide-field trials [median 1.02 (0.99, 1.07) times larger, z=17, and p=0.08]. Significance tests were performed with a Friedman $\chi^{2}$ with post hoc Bonferroni-corrected Wilcoxon signed-rank tests on the raw widths (significant at p<0.0083). Here, n=12 cells from 12 slices from four mice. Friedman $\chi^{2}=26$, $p=9\times 10^{-6}$ (Fig. 5).

In total, our analyses reveal that deconvolution improves both axial and lateral signal localization, but decreases temporal signal SNR compared to synthetic refocusing, with both effects intensifying with increasing iteration number.

### 3.5 Temporal signal SNR is Unaffected by Light-Field Imaging

We measured the SNR for paired wide-field and refocused light-field imaging trials in the same cells. For 8/12 trials, we included a correction factor due to a misalignment in the LFM as discussed in Sec. 2.3.3. The SNR did not change significantly between the light-field and wide-field cases (Wilcoxon signed-rank test, n=12 cells from 12 slices and four mice, z=32, p=0.6), with a median light-field SNR of 8.4 (5.2, 11.4) and a median wide-field SNR of 10.0 (7.6, 11.9) (Fig. 5). We measured the SNR from equivalent pixel areas in wide-field and light-field image series to compare the two techniques. This potentially advantages light field over wide field, as more intricate ROIs can be used with the smaller wide-field pixels, reducing signal spatial averaging and increasing signal size. We expect this effect to be small for the somatic ROIs we used, as the pixels external to the ROIs typically still contained scattered or dendritic voltage signals, and so the increased granularity of the wide-field ROIs would not provide a large SNR advantage. Furthermore, this disadvantage is greatly ameliorated in more recent LFM implementations,^57^^,^^59^^,^^61^ which typically have better lateral resolution, especially at the LFM focal plane.

## 4 Discussion

We have shown that LFM enables 3-D subcellular GEVI imaging of somatic and dendritic structures. We demonstrated that LFM enables simultaneous imaging of axially separated dendrites, overcoming a key limitation of wide-field imaging. We further showed that functional voltage signals from dendrites could be axially resolved at different depths. This finding is the key to demonstrating LFM’s utility for studies of dendritic integration or synaptic mapping.

We compared how synthetic refocusing and deconvolution-based reconstruction techniques perform with respect to spatial signal localization and temporal SNR. Synthetic refocusing is computationally simple and can be used to process light fields online, during an experiment, or post hoc. Refocusing features have better temporal signal SNR but poorer lateral and axial confinement compared to deconvolution. Deconvolution has two major disadvantages: computational cost and noise amplification. As the LFM PSF is not shift invariant, it is described by a five-dimensional matrix, complicating reconstruction. The periodicity it displays under lateral shifts by integer multiples of the microlens pitch, however, enable deconvolution to be performed efficiently using FFT-based convolutions. Despite this, even small increases in lateral sampling in the deconvolved volume increase the computational cost of reconstruction drastically. Reconstructing $n_{z}z$ planes in a volume with a lateral increase in sampling over the native LFM sampling of m requires $2\times n_{z}\times m^{2}$ 2-D convolutions per iteration, precluding online image processing. Second, both RL and ISRA tend to amplify noise in their outputs due to their lack of regularization.^82^ This noise may be acceptable when imaging high-SNR calcium signals, however, it can dominate small, dim voltage signals. Incorporating regularization into the deconvolution approaches to suppress noise overfitting could also ameliorate deconvolution’s effects on temporal SNR. The iteration analysis described in Secs. 3.2 and 3.4 should aid future studies using early stopping of the deconvolution scheme as implicit regularization, as they provide a quantitative measure of the gains and losses at different regularization levels.

For this study, we used the original LFM design, which has since been improved upon significantly by multiple groups.^57^^–^^61^ These advances variously improve the spatial resolution and decrease the computational cost of deconvolution; however, the basic image formation concept remains the same in all the new modalities. The results in this study are likely applicable to voltage imaging with these newer light-field modalities as the reconstruction approaches are similar. In this study, we imaged VSFP-Butterfly 1.2, an older generation probe. GEVI technology has advanced dramatically recently, greatly increasing their sensitivity, and with these new sensors, noise amplification due to deconvolution in the light-field volume reconstruction may become less significant. Although VSFP-Butterfly 1.2 exhibits lower sensitivity than several recently reported probes,^20^^,^^84^^–^^88^ we were able to express it sparsely and strongly to enable single-cell GEVI imaging without somatic restriction, which would preclude study of subcellular signals.^68^^,^^69^ The slow kinetics of the probe used in this study also enabled resolution of action potentials at 100  frames/s without severe aliasing. Although we could resolve single-sweep signals, signal averaging was required to resolve smaller dendritic signals with adequate SNR. With a more recent GEVI, dendritic processes could likely be resolved in single sweeps.

Newer voltage sensors cannot be immediately combined with LFM, however, as they require much faster sampling rates, typically between 500 and 1000 Hz. Megapixel cameras with 1-kHz full-frame readout rates are therefore needed to fully exploit these newer voltage indicators. Current scientific CMOS (sCMOS) cameras such as the one used in this study can achieve these imaging rates by reducing the FOV to a small central strip of the image sensor. This, however, is particularly detrimental to LFM compared to wide-field imaging as the LFM PSF spreads information about each point widely across the image sensor for objects away from the focal plane. If only a small strip of the sensor is imaged, SNR will be greatly degraded as light is lost outside of this reduced FOV. Limiting the camera to 128-pixel rows to achieve a frame rate of 1.6 kHz would reject <50% of all fluorescence from objects further than 35  μm from the focal plane. This lost light would also be unavailable for the reconstruction, resulting in degraded resolution. This drastically limits the applicability of reducing the FOV for increased frame rate, common with sCMOS cameras.

We anticipate that this issue will be steadily ameliorated as faster sCMOS sensor technology is developed.

A second issue arises with newer, faster GEVIs due to their requirement for much faster frame rates. Deconvolving individual frames with these sensors would require a drastic increase in computational resources and is likely untenable. Source extraction approaches have been developed for static light-field images^89^ and light-field calcium imaging time series,^65^^,^^90^ which do not involve deconvolution of every frame. In their current form, however, these are unsuitable for reconstruction of subcellular light-field voltage imaging time series as they leverage the temporal and/or spatial characteristics of neuronal calcium imaging as reconstruction priors. These priors, such as somatic signal localization or sparse temporal activity, are not as applicable to subcellular voltage imaging signals, which are smaller, less temporally sparse and arise from more morphologically intricate structures than neuronal somata. Development of more sophisticated reconstruction algorithms is nevertheless important, especially as previous efforts have enabled imaging deeper in scattering tissue than was possible in this study.

Finally, in this study, we compared the SNR between refocused LFM volumes and matched wide-field traces and found they did not differ significantly. This is expected, as apart from light losses at the MLA, which are <15% according to the manufacturer, there are no significant losses of SNR to shot noise between WFM and LFM. LFM’s ability to resolve voltage signals from different axial planes without repeated physical refocusing and time-series acquisition can drastically reduce imaging time and bleaching. Together these results have the potential to motivate further work and widespread application of LFM to voltage imaging owing to light-field’s high photon budget and ability to resolve neurons in three spatial dimensions.

## Code, Data, and Materials Availability

The datasets and code generated for this study are available on request to the corresponding author.
